# Effects of Melatonin Pre- and Post-Drought Treatment on Oxidative Stress Markers and Expression of Proline-Related Transcripts in Young Wheat Plants

**DOI:** 10.3390/ijms252212127

**Published:** 2024-11-12

**Authors:** Zornitsa Katerova, Dessislava Todorova, Irina I. Vaseva, Elena Shopova, Margarita Petrakova, Martin Iliev, Iskren Sergiev

**Affiliations:** Institute of Plant Physiology and Genetics, Bulgarian Academy of Sciences, 1113 Sofia, Bulgaria; zornitsa@bio21.bas.bg (Z.K.); dessita@bio21.bas.bg (D.T.); vaseva@bio21.bas.bg (I.I.V.); kostei@abv.bg (E.S.); margarita.p02@abv.bg (M.P.); martin.vl.iliev@gmail.com (M.I.)

**Keywords:** wheat, drought, melatonin, stress markers, gene expression analyses

## Abstract

Wheat can tolerate a mild water deficit, but prolonged drought causes a number of detrimental physiological changes resulting in a substantial decrease in productivity. The present study evaluates the potential of the natural plant growth regulator melatonin to alleviate the negative effects of moderate drought in two Bulgarian winter wheat cultivars at the early vegetative stage. Melatonin doses of 75 µM were root-supplemented 24 h before or after the stress period. The levels of several biometric parameters, osmolyte content and stress indicators as well as the expression of genes coding for key enzymes of the proline biosynthesis pathway were analyzed in leaves at the end of the drought stress and after two and four days of recovery. Applied alone, melatonin did not exert significant effects on most of the monitored parameters. Water deprivation negatively affected seedlings’ fresh weight and water content and increased the stress markers and osmolyte levels. These were accompanied by a high accumulation of *TaP5CS* and *TaP5CR* transcripts coding for the enzymes Δ-pyrroline-5-carboxylate synthase and Δ-pyrroline-5-carboxylate reductase, respectively. The effect of melatonin in reducing drought stress was similar whether applied before or after exposure, though slightly more effective when used as a pre-treatment.

## 1. Introduction

Plants are constantly exposed to various abiotic and biotic stress factors. Increasing alerts of prolonged drought events in many regions around the world caused by the global climate change pose a serious risk to crop performance and productivity. Physiological drought occurs due to the unavailability of soil water for plants. Additionally, plants can experience physiological drought caused by a lack of water due to increased soil salinity or high air temperature [1]. A water deficit can occur at any stage of plant development, negatively affecting the processes of germination, growth and reproduction, causing dramatic morphological, biochemical, physiological and molecular changes. Scarce water availability is one of the stress factors directly leading to a significant reduction in productivity of agricultural crops [2,3,4].

Almost all aspects of plant development are affected by drought. A lack of water negatively impacts the plants’ sprouting, vegetative growth and development and their ability to form reproductive organs. A drought-caused decrease in crop yield is related to the disruption of key biochemical and physiological processes. Plant growth in less-than-optimal conditions causes the accumulation of harmful reactive oxygen species (ROS) within the cellular compartments, which is a common response to different types of stress including drought. Although ROS form as a product of normal plant metabolism, the over-accumulation of free radicals may trigger chain oxidation processes that result in the formation of lipid peroxides, causing damage to biomembranes [3,5]. This disturbs basic physiological functions and negatively impacts the homeostasis that sustains optimal growth and development. A common response of plants under stress conditions is the activation of the antioxidant defense system, which includes antioxidants of an enzymatic and a non-enzymatic nature that scavenge ROS. In addition, plant cells accumulate different types of compatible solutes, such as proline, reducing sugars, polyols and other organic and inorganic osmolytes [2,4]. These enable maintenance of cell turgor and biomembrane integrity under drought-stress conditions. Integrated metabolomic and transcriptomic analyses also reveal that osmotic regulation processes and ROS scavenging mechanisms are among the most important characteristics involved in the drought tolerance of wheat [6]. When the stress “dose” does not exceed the adaptive mechanisms of plants, they are able to successfully overcome the external adversities via the mobilization of various defense mechanisms. The natural defense systems of plants could require fortification under prolonged stress conditions, and the application of biostimulants could provide such efficient and environmentally friendly support. Their use for the improvement of plant tolerance to drought relies on the activation of intrinsic mechanisms that control cellular metabolism. The stress-protective effect of biostimulants has been linked to the upregulation of various molecular components that facilitate plant protective reactions to overcome the damages induced by external stress factors [1,7,8,9]. The efficiency of biostimulants as stress-protective agents varies tremendously, and it depends on the environmental conditions, the crop genotype and the developmental stage. However, their use in agriculture over the last few years is increasing, as they have been proven to be environmentally safe, which aligns with the policy to develop sustainable production of clean and healthy food. In addition, biostimulant formulations might be applied as a preventive measure before expected stress, during stress or after stress as agents promoting plant recovery [10]. Some of them contain physiologically active amounts of different phytohormones, plant growth regulators or other biologically active compounds that interfere with stress-protective reactions. Phytohormones and plant growth regulators control a variety of physiological activities tuning multiple cellular processes in plants. Among them, ABA, auxins, brassinosteroids, cytokinins, ethylene, gibberellins, jasmonates, salicylic acid, strigolactones and melatonin are known to participate in the regulation of plant reactions to abiotic stresses including drought tolerance [2,3,4,11,12,13].

Plants naturally produce N-acetyl-5-methoxytryptamine, also known as melatonin, which has been proven to have the ability to stimulate and regulate plant development in response to a variety of environmental stressors [14,15]. Since it is a non-toxic biomolecule, its strategic utilization could increase plants’ ability to withstand diverse stress factors [16]. Research has demonstrated that melatonin has a beneficial effect on the metabolism and physiology of stressed plants, as evidenced by reduced levels of certain stress indicators, such as malondialdehyde (MDA) and cell electrolyte leakage (EL) [16]. It has been suggested that it scavenges reactive oxygen species (ROS), has a stress-signaling role, cooperates with phytohormones and controls a variety of metabolic events in plants [17]. Recent studies report that melatonin alleviates drought stress in various crops by influencing multiple stress-response mechanisms [18,19,20]. Overall, the data derived in these studies offer new insights into the complex mechanisms through which melatonin boosts crop resilience to drought stress, indicating that its protective effect is linked to modulating the expression of different transcription factors and plant hormone signaling cascades.

Data reporting on the application of melatonin as a biostimulant in wheat after exposure to stress (post-treatment) are limited [21,22]. The results on the physiological status of wheat plants following melatonin pre-treatment and post-treatment after drought could be valuable for farmers who may not be aware of impending drought conditions. The current study’s objective is to compare the physiological responses to melatonin administration in two Bulgarian wheat cultivars (cv. Gines and cv. Fermer) varying in their drought tolerance. The selection of the cultivars was based on earlier research on their resistance to drought: cv. Fermer was reported to be more sensitive than cv. Gines [23,24]. This observation was additionally confirmed by phylogenetic analyses of 117 modern European bread wheat genotype varieties (78 of which have Bulgarian origin) [25]. The evaluation of melatonin capacity as a drought-stress-alleviating compound was performed by monitoring malondialdehyde (MDA), electrolyte leakage (EL), hydrogen peroxide (H_2_O_2_), the content of total osmolytes, reducing sugars, free proline and the gene expression of two key enzymes of the proline biosynthesis pathway (*delta-1-pyrroline-5-carboxylate synthase*, *P5CS* and *pyrroline-5-carboxylate reductase*, *P5CR*).

Taken together, our results could extend the knowledge of melatonin action as a biostimulator and outline some limitations concerning diverse tolerance of the used cultivars.

## 2. Results

### 2.1. Growth Parameters

**Figure 1:**
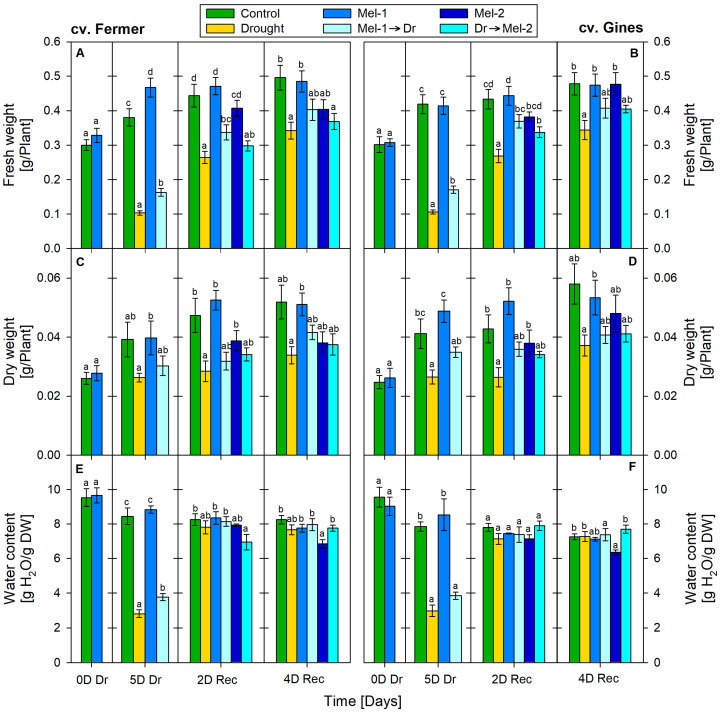
Growth parameters: fresh weight (**A**,**B**), dry weight (**C**,**D**) and water content (**E**,**F**) in cv. Fermer (**A**,**C**,**E**) and cv. Gines (**B**,**D**,**F**) at 0 day of drought (0D Dr), at the 5th day of drought (5D Dr), at the 2nd day of recovery (2D Rec) and at the 4th day of recovery (4D Rec). The abbreviations within the legend denote as follows: Mel-1—melatonin control before drought, Mel-2—melatonin control after drought, Mel-1→Dr—melatonin pre-application before drought, Dr→Mel-2—melatonin post-application after drought. Different letters within each panel section indicate significant differences. Error bars designate standard error.

Severely decreased fresh weight (FW) was registered after 5 days of drought (5D Dr): decreased by 70% (from 0.380 g average weight of the controls to 0.103 g average weight of the stressed individuals) in cv. Fermer (Figure 1A) and by 75% (0.419 g and 0.106 g, respectively, in the control and in the drought-stressed group) in cv. Gines (Figure 1B). That tendency gradually receded and dropped to 40% on the 2nd day of recovery (2D Rec) and to 30% on the 4th day of recovery (4D Rec) for both cultivars. The FW increased slightly (by 20%) in the plants that received melatonin application (Mel-1) at 5D Dr in cv. Fermer. The application of melatonin prior to drought (Mel-1→Dr, pre-treatment) mitigated the FW loss due to drought, and the detected decrease was 60% (0.163 g average weight for cv. Fermer and 0.171 g average weight for cv. Gines at 5D Dr) and 20% (at 2D Rec in cv. Fermer) and reached the respective control values by the end of the experimental period. The application of melatonin after drought (Dr→Mel-2, post-treatment) showed a similar trend, matching that of Mel-1→Dr only in cv. Gines. The FW parameter showed improvement at the end of the experiment. The application of Dr→Mel-2 in cv. Fermer did not show a considerable alteration in FW compared to drought.

Drought decreased dry weight (DW) in both cultivars by approximately 40% (average control: 0.039 g and 5D Dr: 0.026 g for cv. Fermer; average control: 0.041 g and average 5D Dr: 0.026 g for cv. Gines; Figure 1C,D). The DW was not affected by melatonin pre-treatment application alone (Mel-1) at 0D Dr, 5D Dr and 4D Rec in cv. Fermer. For the same experimental groups (Mel-1) of the Guines variety, we found a slight increase in the DW up to 2D Rec. The combination of stress and melatonin (pre- and post-treatment) diminished the negative impact on DW in both tested wheat cultivars.

Decreased water content was documented at 5D Dr: decreased by 70% in cv. Fermer (Figure 1E) and by 60% in cv. Gines (Figure 1F). The Mel-2 application alone slightly reduced water content: reduced by 20% and by 10% at 4D Rec in cv. Fermer and cv. Gines, respectively. Water content was not altered significantly during the entire period of recovery (2D Rec and 4D Rec) after both of the combined treatments (Mel-1→Dr and Dr→Mel-2).

### 2.2. Stress Markers Content

**Figure 2:**
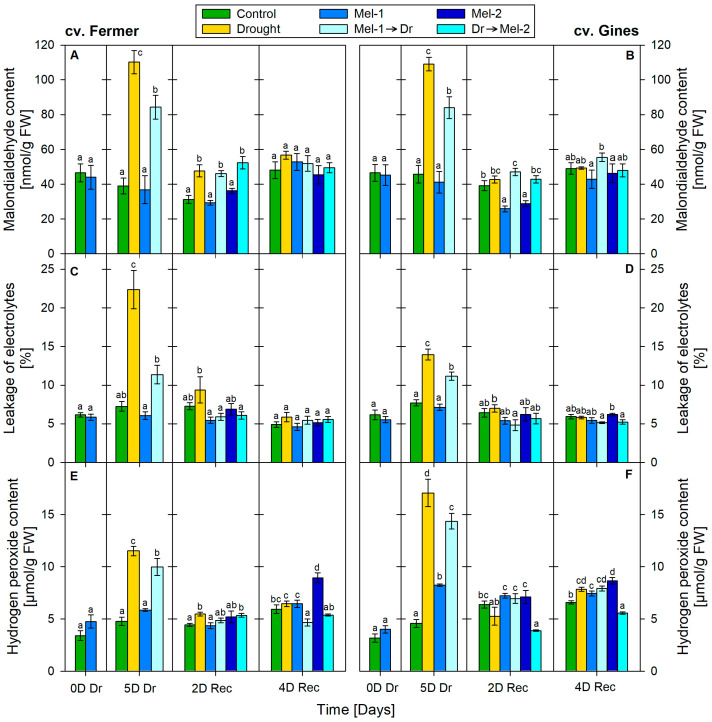
Content of stress markers malondialdehyde (**A**,**B**), leakage of electrolytes (**C**,**D**) and hydrogen peroxide (**E**,**F**) in cv. Fermer (**A**,**C**,**E**) and cv. Gines (**B**,**D**,**F**) at 0 day of drought (0D Dr), at the 5th day of drought (5D Dr), at the 2nd day of recovery (2D Rec) and at the 4th day of recovery (4D Rec). The abbreviations within the legend denote as follows: Mel-1—melatonin control before drought, Mel-2—melatonin control after drought, Mel-1→Dr—melatonin pre-application before drought, Dr→Mel-2—melatonin post-application after drought. Different letters within each panel section indicate significant differences. Error bars designate standard error.

After 5 days of drought, the MDA level was increased by 180% (to 110.16 nmol/g FW) in cv. Fermer (Figure 2A) and by 140% (to 109.05 nmol/g FW) in cv. Gines (Figure 2B) as compared to the control. Later, at 2D Rec, it was still 50% higher than the control in cv. Fermer, while in cv. Gines, it was close to the control level. The MDA content decreased more significantly due to melatonin treatment alone only in cv. Gines: decreased by 30% at the 2nd day of recovery. The measured MDA levels in the Mel-1→Dr experimental group (cv. Fermer: 84.29 nmol/g FW, cv. Gines: 84.02 nmol/g FW) were below the ones that were measured in the drought-treated (cv. Fermer: 110.16 nmol/g FW, cv. Gines: 109.05 nmol/g FW). At the 2nd day of recovery, MDA levels remained high after application of Dr→Mel-2 in cv. Fermer but not in cv. Gines.

Melatonin application alone (Mel-1 and Mel-2) did not cause changes in the electrolyte leakage (EL) levels in both cultivars. The water deficit significantly increased this stress marker at the 5th day of drought: increased by 210% for cv. Fermer (Figure 2C) and by 80% for cv. Gines (Figure 2D). The combined application of Mel-1→Dr considerably lessened drought-induced EL alterations at the 5th day of drought: the EL was only 60% higher than the control for cv. Fermer and 50% for cv. Gines. Both cultivars showed quick recovery in relation to this parameter, and at 2D Rec, it tended to reach the controls. It should be noted that EL remained higher in cv. Fermer than that measured in cv. Gines. The combined application of Dr→Mel-2 did not show significant differences in EL compared to the control.

Drought increased H_2_O_2_ concentrations by 140% (average value of 4.79 µmol/g FW for the control and average of 11.52 µmol/g FW measured in the stressed individuals) in cv. Fermer (Figure 2E) and by 270% (from 4.58 µmol/g FW measured in the control to 17.07 µmol/g FW in the stressed plants) in cv. Gines (Figure 2F). At the 2nd day of recovery, a 20% increase in H_2_O_2_ in cv. Fermer was detected. It was found that, at the 5th day of drought, hydrogen peroxide levels were also raised by 80% after Mel-1 application (8.25 µmol/g FW) in cv. Gines. A similar effect was observed for Mel-2 at the 4th day of recovery, which increased H_2_O_2_ by 50% and 30% in cv. Fermer and cv. Gines, respectively. After the combined application of Mel-1→Dr, it was increased by 110% (9.98 µmol/g FW) in cv. Fermer and by 210% (14.36 µmol/g FW) in cv. Gines at the 5th day of drought. However, at the end of the experimental period, pre-treatment with melatonin (Mel-1→Dr) had contrasting effects on H_2_O_2_ levels for both cultivars: a decline (20%, from 5.96 to 4.67 µmol/g FW) in cv. Fermer and an increase (20%, from 6.60 to 7.94 µmol/g FW) in cv. Gines samples. Regarding the post-treatment with melatonin (Dr→Mel-2), at the 2nd day of recovery, there was an opposing trend of H_2_O_2_ alteration in both cultivars (20% increase in cv. Fermer and 40% reduction in cv. Gines). At the 4th day of recovery, only a 20% decrease was found in cv. Gines.

### 2.3. Compatible Solutes

**Figure 3:**
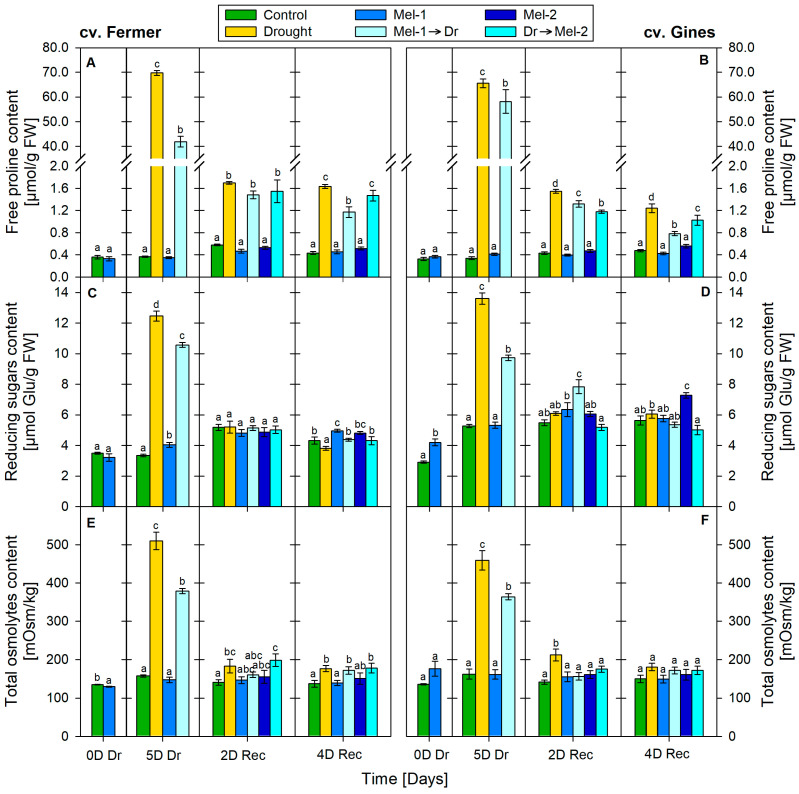
Content of compatible solutes: free proline (**A**,**B**), reducing sugars (**C**,**D**) and total osmolytes (**E**,**F**) in cv. Fermer (**A**,**C**,**E**) and cv. Gines (**B**,**D**,**F**) at 0 day of drought (0D Dr), at the 5th day of drought (5D Dr), at the 2nd day of recovery (2D Rec) and at the 4th day of recovery (4D Rec). The abbreviations within the legend denote as follows: Mel-1—melatonin control before drought, Mel-2—melatonin control after drought, Mel-1→Dr—melatonin pre-application before drought, Dr→Mel-2—melatonin post-application after drought. Different letters within each panel section indicate significant differences. Error bars designate standard error.

Drought severely increased proline content. At the 5th day of drought, it was 190-fold higher than the controls in both cultivars (cv. Fermer: from 0.37 to 69.77 µmol/g FW; cv. Gines: from 0.34 to 65.58 µmol/g FW; Figure 3A,B). After restoring the normal irrigation, the proline levels quickly dropped but remained 2–3-fold higher than the respective controls (Figure 3A,B). Proline content was not altered significantly due to melatonin application alone (Mel-1 and Mel-2) during the entire experimental period. Melatonin pre-treatment (Mel-1→Dr) significantly reduced proline in cv. Fermer as compared to the drought-treated plants at 5D Dr (from 190-fold—69.77 µmol/g FW—to 110-fold—41.83 µmol/g FW). A similar trend was also found, to a lesser extent, in cv. Gines (from 190-fold—65.58 µmol/g FW—to 170-fold—58.14 µmol/g FW). The observed trends of maintenance of lower proline levels in melatonin pre-treated seedlings were consistently observed throughout the entire experimental period. A similar, although less pronounced, effect was detected after post-treatment with melatonin (Dr→Mel-2) in cv. Gines.

Increased levels of reducing sugars (RS), by 270% (from 3.34 to 12.48 µmol Glu/g FW in cv. Fermer, Figure 3C) and by 160% (from 5.26 to 13.61 µmol Glu/g FW in cv. Gines, Figure 3D), at the 5th day of drought were documented in the plants subjected to water deprivation only. At the end of the experiment, RS content was below the control by 20% in cv. Fermer, while in cv. Gines, it was near the control. Melatonin application alone slightly raised the concentrations of RS as follows: 40% (0D Dr, Mel-1, cv. Gines), 20% (5D DR, Mel-1, cv. Fermer) and 20% (4D Rec, Mel-2, cv. Gines). Only the pre-treatment (Mel-1→Dr) but not the post-treatment (Dr→Mel-2) caused a significant reduction in RS levels as compared to the group subjected only to drought.

A water deficit increased the total osmolyte content (Figure 3E,F) by 220% (510.24 mOsm/kg) and by 180% (459.75 mOsm/kg) (at the 5th day of drought) in cv. Fermer and cv. Gines, respectively. After restoring the normal irrigation regime, the concentration of osmolytes tended to decrease but remained higher than the control by 30% in cv. Fermer and by 20% in cv. Gines at the 4th day of recovery. The melatonin treatment alone did not alter the total osmolyte content. Compared to drought, the content of osmolytes was less increased after the pre-treatment with melatonin (Mel-1→Dr), and it was 140% (379.01 mOsm/kg) and 125% (364.20 mOsm/kg) above the control at the 5th day of drought in cv. Fermer and cv. Gines, respectively. Regarding the post-treatment with melatonin (Dr→Mel-2), a noticeable diminished negative effect on the drought-induced accumulation of osmolytes was detected only in cv. Gines at the 2nd day of recovery.

### 2.4. Transcript Profiling of Genes from the L-Proline Biosynthesis Pathway in Samples Derived from Wheat Plants Subjected to Drought or Combination with Melatonin

**Figure 4:**
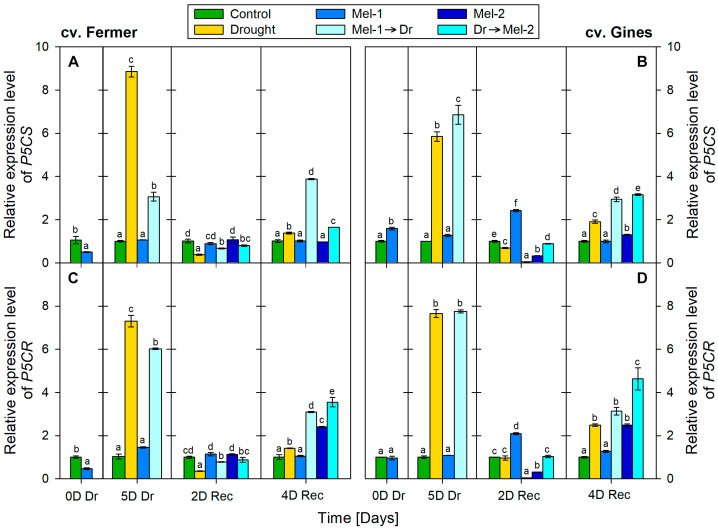
Relative expression levels of *T. aestivum delta-1-pyrroline-5-carboxylate synthase* (*P5CS*) (**A**,**B**) and *pyrroline-5-carboxylate reductase* (*P5CR*) (**C**,**D**) genes in cv. Fermer (**A**,**C**) and cv. Gines (**B**,**D**) leaves from plants. The abbreviations within the legend denote as follows: Mel-1—melatonin control before drought, Mel-2—melatonin control after drought, Mel-1→Dr—melatonin pre-application before drought, Dr→Mel-2—melatonin post-application after drought. Different letters within each panel section indicate significant differences. Error bars designate standard error.

We examined the expression of two important genes coding key enzymes of proline biosynthesis—delta-1-pyrroline-5-carboxylate synthase (P5CS, Figure 4A,B) and pyrroline-5-carboxylate reductase (P5CR, Figure 4C,D). These enzymes catalyze the rate-limiting step (P5CS) and the final step of L-proline biosynthesis from glutamate (P5CR), respectively [26].

**Figure 5:**
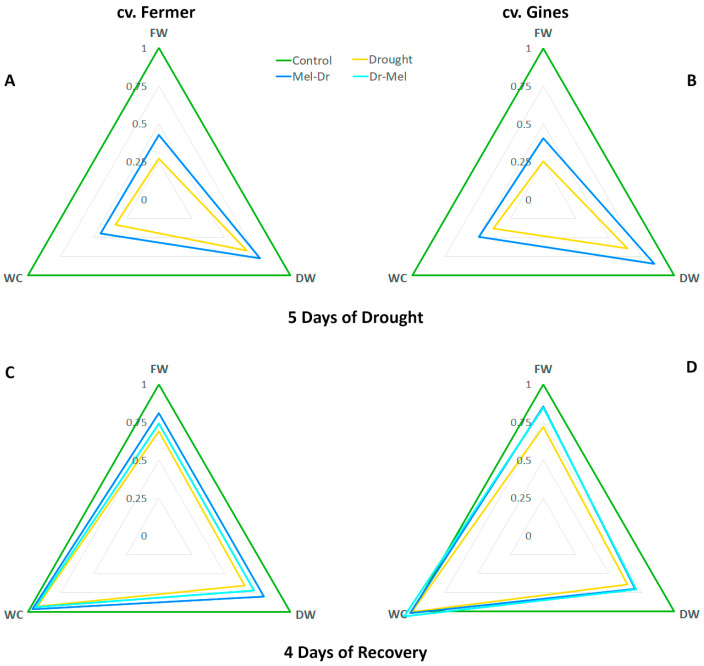
Spider plot of the growth traits fresh weight (FW), dry weight (DW) and water content (WC) at the 5th day of drought (**A**,**B**) and 4th day of recovery (**C**,**D**) in wheat cv. Fermer (**A**,**C**) and cv. Gines (**B**,**D**). The values of the parameters are normalized to the control.

Initially (5D Dr), drought increased transcript abundance of P5CS: increased 8-fold in cv. Fermer (Figure 4A) and 5-fold in cv. Gines (Figure 4B). Then, at 2D Rec, the expression was downregulated by 60% and by 30% as compared to the respective control of each cultivar. At the final measurement point (at 4D Rec), the expression was upregulated by 40% and by 90% in cv. Fermer and cv. Gines, respectively. At 0D Dr, the expression of the P5CS gene decreased by 50% in cv. Fermer but increased by 60% in cv. Gines due to melatonin treatment alone (Mel-1). However, starting from 5D Dr, the expression of P5CS was influenced by melatonin alone application (Mel-1 and Mel-2) only in cv. Gines. Mel-1 increased the transcript abundance by 140% at 2D Rec, but then it reached control values at 4D Rec. The post-stress treatment (Mel-2) initially downregulated the expression of P5CS by 70% at 2D Rec, but then (at 4D Rec) an increase in the transcript abundance (by 30%) was registered. Initially (at 5D Dr), the pre-treatment with melatonin (Mel-1→Dr) increased gene expression of P5CS 2-fold (cv. Fermer) and 6-fold (cv. Gines). At the 2nd day of recovery, the transcript abundance dropped by 30% (cv. Fermer) and by 95% (cv. Gines). At the end of the experiment (at 4D Rec), it increased 3- and 2-fold, respectively. Similarly, the expression of P5CS in Dr→Mel-2 plants initially (at 2D Rec) decreased slightly by 20% and by 10%, respectively, in cv. Fermer and cv. Gines, but at the 4th day of recovery, an increase by 60% and 220% was detected.

The P5CR expression (Figure 4C,D) was initially upregulated 7-fold in the drought-stressed plants (5D Dr) in both cultivars. Later on, at the 2nd day of recovery, in cv. Fermer, it was downregulated by 60% as compared to the control, while in cv. Gines, the gene expression maintained control levels. At the end of the experiment (at 4D Rec), it was induced by 40% in cv. Fermer and by 150% in cv. Gines. Significant changes in the gene expression after melatonin alone application (Mel-1) were registered in cv. Fermer at 0D Dr (downregulation by 50%) and in cv. Gines at 2D Rec (upregulation by 110%). At 2D Rec, the post-drought melatonin application (Mel-2) decreased the P5CR expression by 70% in cv. Gines, while in cv. Fermer, the gene expression did not differ from the control. At the 4th day of recovery, Mel-2 increased the transcript abundance by 140% in cv. Fermer and by 150% in cv. Gines.

When compared to the drought-treated-only plants, the melatonin pre-treatment (Mel-1→Dr) markedly decreased the expression of P5CR at the 5th day of drought in cv. Fermer but did not influence the gene expression in cv. Gines. Then, at 2D Rec, the gene expression was decreased by 20% in cv. Fermer and by 96% in cv. Gines. At the 4th day of recovery, P5CR expression was increased 2-fold in both genotypes. Similarly, melatonin post-treatment (Dr→Mel-2) had an effect on P5CR gene expression at the end of the experiment and provoked 3-fold transcript induction in both cultivars.

### 2.5. Spider Plot Presentation of the Studied Parameters Normalized to the Control

**Figure 6:**
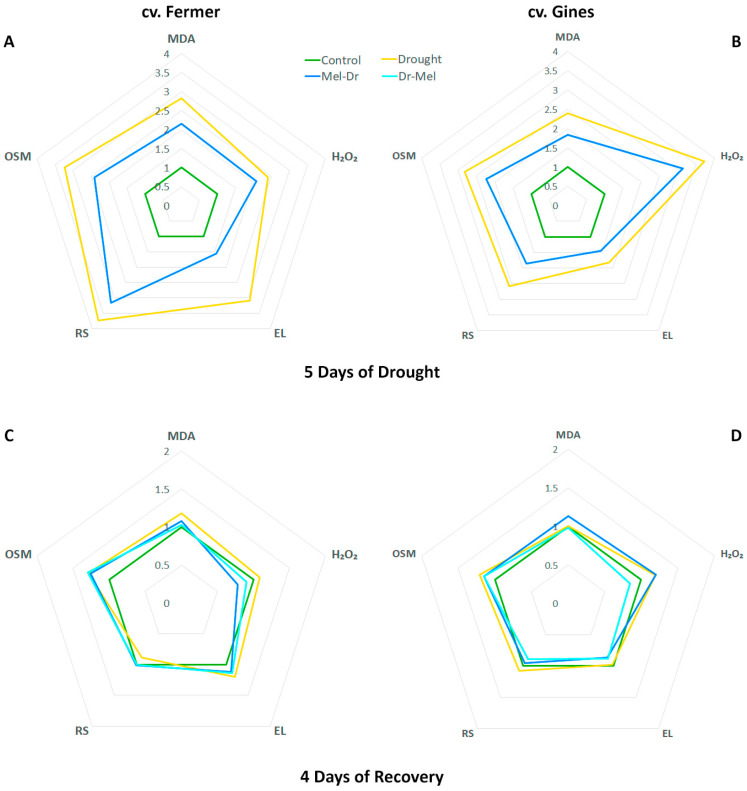
Spider plot of malondialdehyde (MDA), hydrogen peroxide (H_2_O_2_), electrolyte leakage (EL), reducing sugars (RS) and total osmolytes (OSM) at the 5th day of drought (**A**,**B**) and 4th day of recovery (**C**,**D**) in wheat cv. Fermer (**A**,**C**) and cv. Gines (**B**,**D**). The values of the parameters are normalized to the control.

**Figure 7:**
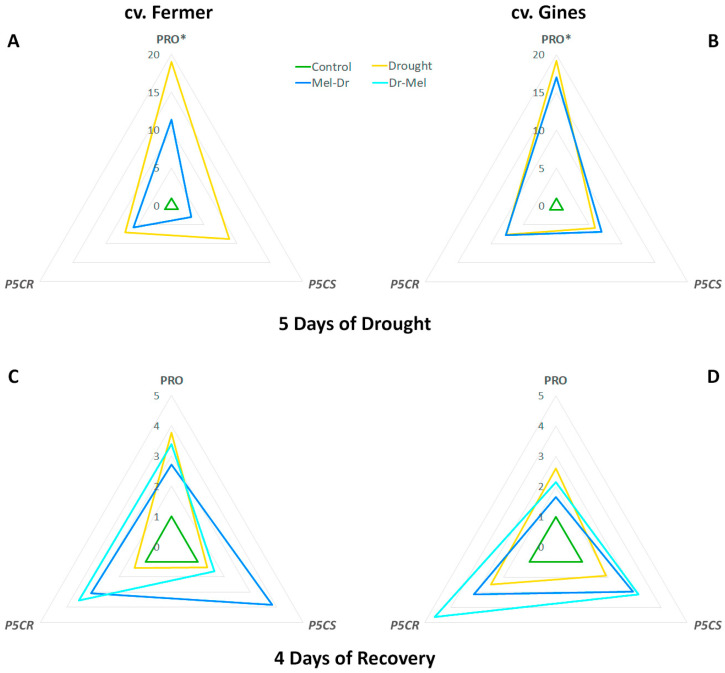
Spider plot of proline (PRO) and expression levels of the proline biosynthesis genes *delta-1-pyrroline-5-carboxylate synthase* (*P5CS*) and *pyrroline-5-carboxylate reductase* (*P5CR*) at the 5th day of drought (**A**,**B**) and 4th day of recovery (**C**,**D**) in wheat cv. Fermer (**A**,**C**) and cv. Gines (**B**,**D**). The values of the parameters are normalized to the control. PRO*: the real ratio at 5 days drought is 10-times higher.

The amplitudes of the alterations in growth traits (Figure 5), stress markers, osmolytes and RS (Figure 6), and proline and the expression profiles of proline-related genes (Figure 7), presented as spider plots at 5D Dr (panels A, B) and 4D Rec (panels C, D), revealed that drought provoked a significant deviation from the physiological levels, which is valid for both cultivars. Both pre-treatment and post-treatment with melatonin reduced the drought-induced changes to some extent. Pre-treatment with melatonin kept the parameters closer to the control during the drought period, which suggests a protective effect against water deprivation. Although Mel-2 was applied shortly after the stress end and acted for a shorter duration, it also helped to mitigate drought damage and supported the affected plants in recovering more quickly.

## 3. Discussion

Stress conditions typically lead to a significant increase in the production of reactive oxygen species (ROS), which can damage plant cell membranes and cause substantial injuries that disrupt normal growth [5].

In the current study, two Bulgarian wheat cultivars were exposed to moderate drought conditions. The design of the model system addresses both the physiological consequences of melatonin pre-treatment (Mel-1) before drought and the effects of post-treatment (Mel-2) after the stress has been terminated by resuming the water supply. To our knowledge, a comparison between the physiological consequences of melatonin pre-treatment and post-treatment has not been reported before, especially regarding the studied Bulgarian wheat cultivars, which were proven by phylogenetic analyses to differ from the Central and Western European varieties [25].

A common approach for evaluating the stress effects on plant cells involves measuring the levels of stress biomarkers, such as MDA, hydrogen peroxide and electrolyte leakage (EL). Hydrogen peroxide and MDA are often used as indicators of oxidative stress in plants. EL and MDA are also regarded as markers for membrane deteriorations [16]. The literature review revealed that, in plants, including wheat cultivars, grown in non-stressful environments [27,28,29], melatonin treatment did not provoke appreciable changes in stress-related biomarkers [30,31,32,33]. In the present study, melatonin application was found to maintain stable low levels of stress-related biomarkers associated with membrane integrity (MDA and EL) and proline. Previously [34], we found no notable changes in some stress biomarkers (EL, MDA, proline) of the same wheat cultivars after melatonin application in the early vegetative stage; now, we update this research with additional evidence for important ROS and also with the lack of disruption in growth traits. The moderate induction of H_2_O_2_ due to melatonin application was better expressed in the more drought-tolerant cv. Gines, and this could be linked to the signaling function of hydrogen peroxide [5]. The stable growth parameters (FW and DW) due to Mel-1 and Mel-2 application for both wheat cultivars additionally demonstrated the beneficial effects of Mel on plant growth. The slight decrease in water content following Mel-2 treatment may be related to melatonin’s interference with stomatal conductance [33].

The transient variation in proline-synthesis gene expression levels (*TaP5CS* and *TaP5CR*) due to Mel-1 and Mel-2 did not seem to affect proline levels in the different experimental groups. Similarly, Chen et al. [35] and Vendruscolo et al. [36] reported that proline levels were not affected in *VyP5CR A. thaliana* overexpression lines and transgenic wheat overexpressing *P5CS* under control conditions. However, Ma et al. [37] observed increased proline levels in non-treated *35S*::*TaP5CR* plants. The minor increase in reducing sugars due to Mel-1 (5D Dr and 4D Rec) in cv. Fermer and after both Mel-1 (0 D Dr) and Mel-2 (4D Rec) in cv. Gines did not affect the total osmolyte content but corresponded well with the signaling role of soluble sugars [38,39]. The earlier induction in reducing sugars due to Mel-1 treatment for cv. Gines as compared to cv. Fermer might be explained by their different drought tolerance [23,24].

The observed negative effects in the growth traits FW, DW and water content (Figure 1 and Figure 5) and stress markers EL, MDA and H_2_O_2_ (Figure 2 and Figure 6) at the 5th day of drought due to the water deficit are in line with other publications [40,41,42,43]. In the present study, we applied moderate drought to outline the possible deviations in physiological responses to stress that are tailored to the respective genotype. The cultivar-specific accumulation of H_2_O_2_ and the rise in membrane damage stress markers (MDA and EL) confirm the occurrence of oxidative stress events at 5D Dr that are differently manifested depending on the drought tolerance of the cultivars (Figure 2 and Figure 6) [23,24]. The higher increase in H_2_O_2_ concentration in cv. Gines than in cv. Fermer at 5D Dr together with lower levels of membrane damage stress markers (EL, MDA) might be related to the probable activation of the antioxidant machinery, as the monitored ROS quickly reached (already at 2D Rec) the control levels. A similar explanation could be valid for the milder increase in reducing sugars observed in cv. Gines as compared to cv. Fermer, as both soluble sugars and proline have a radical scavenger role and the generation of ample osmolytes is described to be required for tolerant plants [38,44].

Proline was reported to have various functions as not only a carbon and nitrogen source but also as a compatible solute with osmoprotective functions, an antioxidant and ROS scavenger, a molecular chaperone and a signaling molecule [26]. Its high concentrations are often associated with stress tolerance in plants, but the overproduction may lead to toxicity, especially if pyrroline-5-carboxylate (P5C) is amplified [26,45]. Therefore, the severe rise in proline at 5D Dr (Figure 3 and Figure 7) in both cultivars signifies its function as a stress marker. This is in line with the notable reduction in the growth parameters (Figure 1 and Figure 5), indicative of impaired homeostasis. We observed greater fluctuations in the expression of proline biosynthesis genes due to drought stress, which do not correspond with the modest decline in proline levels over time. One possible explanation is feedback regulation, which is likely more evident in gene expression than in the final proline product, particularly given the significant improvement in water content over time during recovery.

Other authors [46] also reported increased proline levels due to drought in wheat, which was not associated with enhanced *P5CS* gene expression. Our results correspond well with the suggestion of Chen et al. [35] that there probably are species- and stress-specific variations in the function and regulation of P5CR. In addition, beyond the reported presence of two or more copies of *P5CR* in wheat [37], two *P5CS* isoforms with different function and localization were described in various plants [47,48]. Our data (Figure 2 and Figure 6) are in accordance with the observation of enhanced osmotic tolerance in transgenic Arabidopsis overexpressing *VyP5CR* or *TaP5CR*, which correlated well with the decrease in MDA content [35,37]. The observed difference in proline synthesis gene expression in both cultivars (more enhanced levels of *P5CS* in cv. Fermer (Figure 4A,C) but *P5CR* in cv. Gines (Figure 4B,D) could be another element of the cultivar-specific drought-coping mechanisms. The reports that stress-induced proline synthesis in transgenic plants overexpressing *P5CS* resulted in drought tolerance [36] and that induced proline and *P5CS* gene expression were observed in drought-tolerant wheat cultivars [48] correspond to the observations made in the present study.

Comparable proline content due to moderate drought stress in sensitive and tolerant wheat cultivars was reported, but differences in the metabolic adjustment concerning accumulation of glucose and fructose were observed [41]. Under drought stress, the accumulation of reducing sugars via breakdown of the storage sugars has osmoregulating and osmoprotective functions, which are key elements for the maintenance of osmotic balance in plants [38,41]. Probably, the higher induction of reducing sugars obtained at 5D Dr in the less-drought-tolerant cv. Fermer as compared with cv. Gines is also part of the different drought management strategies of the two cultivars. Interestingly, the more tolerant cv. Gines had a milder increase in reducing sugars but stable in time as compared to cv. Fermer.

The melatonin pre-treatment (Mel-1→Dr) alleviated, to some extent, the negative growth traits pattern in both wheat cultivars (Figure 1 and Figure 5). Thus, the wheat plants had an opportunity to recover better using the biochemical resource of the available biomass. Similar observations for melatonin pre-treatment before drought stress were reported in various plants, such as wheat, maize, alfalfa, rapeseed, etc. [32,33,49,50,51]. Dai et al. [33] suggested that melatonin pre-treatment could sustain stomatal transport of water and CO_2_, supporting photosynthesis under a water shortage due to the increased stomatal aperture. The melatonin application before drought (Mel-1→Dr) successfully reduced the levels of the examined stress-related biomarkers (MDA, EL, H_2_O_2_) compared to drought (Figure 2 and Figure 6). The stronger positive effect of Mel-1 on EL and proline (especially at 4D Rec) in the drought-tolerant cv. Gines corroborates well with the cultivar‘s better performance under water-limiting conditions and its better capacity to sustain oxidative stress. The attenuating effect of pre-treatment with melatonin (Mel-1→Dr) on ROS observed in cv. Gines could be attributed to the H_2_O_2_ property to act as a signaling molecule that sustains the better physiological status during the recovery stage of the experiment (Figure 6). The inhibition of various antioxidant enzymes that reduce H_2_O_2_ levels or the induction of enzymes that increase H_2_O_2_ levels could not be ruled out [5]. Melatonin pre-treatment (Mel-1→Dr) provokes different RS induction patterns, which corroborates well with the fact that sugars may regulate the expression of numerous genes in plants and, thus, assist with the mitigation of ROS accumulation [38,39]. The sucrose disaccharide, for example, is a source of energy and carbon and can produce some of the sugar-related signaling molecules, such as glucose and trehalose-6-phosphate. The hexose monosaccharide glucose was reported to possess plant hormone-like characteristics, and a disturbance in hexose signaling altered sensitivity to auxin and cytokinin in *Arabidopsis thaliana* [38,39].

When comparing both cultivars, cv. Gines showed a better recovery in relation to drought-induced proline as a stress marker at 4D Rec (Figure 7), which aligns with the observed differences in their drought tolerance. Regarding the outcome of pre- and post-drought treatments with melatonin on proline accumulation, it should be noted that the preliminary application (Mel-1→Dr) showed a better alleviating effect than post-treatment (Dr→Mel-2), which could be explained by the shorter time of melatonin post-treatment to manifest its protective capability. The diverse activation patterns of the proline synthesis transcript levels as a result of melatonin pre- and post-drought treatment (Figure 7) are in line with the suggestion of Chen et al. [35] that stress and species-specific variations in the regulation of proline biosynthesis might exist. We found that the transcripts coding the rate-limiting P5CS have divergent profiles in the two tested varieties (Figure 7). Our data are in accordance with the observation of Vendruscolo et al. [36] that the drought tolerance in transgenic plants overexpressing *P5CS* was mostly due to the activation of oxidative stress-defense mechanisms but not osmotic regulation. The amplitude of deviations in growth traits, stress markers, osmolytes and RS, and proline and the expression profiles of proline-related genes allow for us to speculate that the physiological response in the melatonin-supplemented groups relies on different strategies in the studied cultivars. According to the obtained amplitude of alterations (Figure 6), these response mechanisms seem to be related to osmolyte content including RS in cv. Fermer and H_2_O_2_ in cv. Gines.

In general, the observed drought-stress-attenuating effect of melatonin post-treatment (Dr→Mel-2) was similar to but milder than Mel-1→Dr. Previously, Kurt-Celebi et al. [21] also reported more obvious effects in the preliminary-treated plants than in those treated with melatonin after exposure to gamma radiation. Nevertheless, the authors concluded that both melatonin applications have the capacity to mitigate the adverse γ-ray effects. In our experimental model, even when applied in a later phase, melatonin was capable of mitigating the negative consequences of drought and improving growth parameters compared to wheat plants subjected to drought only (Figure 5, Figure 6 and Figure 7). Our data revealed that melatonin post-drought treatment (Dr→Mel-2) supported plants in regaining FW. Moreover, post-drought treatment was also effective in adjusting the osmolyte, RS and proline contents. This indicates better utilization of the available resources towards improved recovery, particularly in the more drought-tolerant cultivar cv. Gines.

The results obtained give ground to extend further the investigations on pre- and post-drought melatonin application with a focus on photosynthesis and the enzymatic and non-enzymatic antioxidative systems, which will contribute to a better understanding of the potential of melatonin to alleviate the negative drought consequences in wheat plants and to explore the possibilities of its use as an effective tool toward the expanding drought-stress problem.

## 4. Materials and Methods

### 4.1. Plant Material and Treatments

Two Bulgarian wheat cultivars (*Triticum aestivum* L., cv. Fermer and cv. Gines) were used in the experiments. These two varieties were reported to possess different drought tolerance based on the relative water deficit [23] and on the EL, fresh weight and leaf protein content [24]. The seeds were obtained from the Institute of Plant Genetic Resources (Sadovo, Bulgaria). The seeds were sown in pots (Ø9.5 cm, 12 cm height) containing 560 g of soil:sand (3:1) substrate. The soil (leached meadow cinnamon soil, pH 6.2) was obtained from the experimental field of the Institute of Plant Physiology and Genetics near Sofia (Bulgaria). The plants were grown in a growth chamber under the following conditions: photoperiod of 14/10 h with 200 μmol/m^2^/s photon flux density, day/night temperatures of 21 °C/19 °C and 60% relative air humidity. Each treatment set contained six pots with twenty plants per pot. Young wheat plants (17-day-old seedlings with fully developed second leaf—BBCH stage 12) were subjected to a water deficit for 5 days. The drought was implemented by withholding watering, while the control plants were daily irrigated to maintain 75% of the field soil moisture capacity. Following the drought period, the normal irrigation regime was restored, and the plants were left to recover for 4 days. A portion of both the control and drought-exposed plants received a root treatment of 10 mL of a 75 µM aqueous melatonin solution, administered either 24 h before or after the stress period. Samples for analyses were taken from the above-ground part of the plants before drought (0d Dr), at the 5th day of drought (5D Dr) and during the recovery period—2 days after drought (2D Rec) and 4 days after drought (4D Rec). Biometric parameters were measured immediately after harvesting. The contents of the stress markers and osmolytes and the expression of genes related to proline biosynthesis were measured in samples frozen in liquid nitrogen and preserved at −80° C until the analyses.

### 4.2. Biometric Parameters and Electrolyte Leakage Assessment

The fresh weight of above-ground parts was measured immediately after harvesting. The dry weight was measured after drying the same plant material at 110 °C for 48 h in a thermostatic oven. Water content was calculated according to Tounekti et al. [52] using the following formula:

WC = (FW − DW)/DW.

Biomembrane integrity was assessed by measuring the electrolyte leakage according to the procedure described in Cui et al. [40] using a FiveEasy Plus conductivity meter (Mettler-Toledo GmbH, Greifensee, Switzerland) with slight modifications. Briefly, 1 cm-long leaf sections (approximately 40 mg) were put in test tubes containing 45 mL bi-distilled water. Before immersing the plant samples, the conductivity of the bi-distilled water was measured (C_0_). The samples were incubated at 37 °C for 2.5 h, and the initial sample conductivity was measured (C_i_). Then, the samples were incubated for 35 min in a boiling bath and cooled down to room temperature, and the total sample conductivity was measured (C_T_). The relative electrolyte leakage was calculated by the following formula:

REL = (C_i_ − C_0_)/(C_T_ − C_0_).

### 4.3. Content of MDA, H_2_O_2_ and Free Proline

Approximately 300 mg of fresh leaf material was grinded with quartz sand in 4 mL of 1% (*w*/*v*) cold trichloroacetic acid, followed by a 30-min centrifugation at 15,000× *g* at 4 °C. The obtained supernatants were used for measuring the concentrations of free proline, malondialdehyde and hydrogen peroxide.

The reaction mixture for proline measurement consisted of 0.5 mL of supernatant and 0.5 mL 1% trichloroacetic acid along with 1 mL of concentrated acetic acid and 1 mL of ninhydrin reagent [53]. It underwent incubation in boiling water for 1 h, and then, the tubes were placed in an ice bath to stop the reaction. The sample absorbance was measured at 520 nm. A standard curve was used to determine the proline concentration.

The level of lipid peroxidation in the plant tissues was determined by measuring the concentration of MDA as described by Kramer et al. [54]. A half mL of supernatant was mixed with a 1 mL solution prepared with 0.5% thiobarbituric acid in 20% TCA and boiled in a water bath at 100 °C for 45 min. After allowing the mixture to cool on ice and spinning it in a centrifuge for 5 min at 3000 rpm, the optical density was measured at 532 nm and 600 nm. The MDA content was calculated using a molar extinction coefficient of 155 mM^−1^ cm^−1^. For the determination of hydrogen peroxide content, 75 µL supernatant was mixed with 75 µL 1 M KI and incubated for 1 h at room temperature in darkness [55]. Then, the absorbance was read at 390 nm, and the results were calculated by a standard curve prepared with known concentrations of H_2_O_2_.

### 4.4. Content of Osmolytes and Reducing Sugars

The leaf material (approximately 200 mg) was ground in 4 mL of bi-distilled water and was centrifuged for 30 min at 15,000× *g* (4 °C). The supernatant was used to analyze the reducing sugar content (RSC), according to the procedure of Gonçalves et al. [56] with slight modifications, and for measuring the total osmolytes according to [57]. All test tubes contained 500 μL of dinitrosalicylic acid reagent (prepared with 3,5-dinitrosalicylic acid, potassium sodium tartarate tetrahydrate and NaOH) and 500 μL of supernatant or distilled water (blank). The reaction mixture was incubated for 5 min at 100 °C and then cooled on ice. Five mL of distilled water was added to each tube, and the absorbance was read at 540 nm. The RSC was calculated by a standard curve prepared with known concentrations of glucose.

Total osmolyte content was measured with a cryoscopic freezing-point osmometer, Osmomat 3000 (Gonotec GmbH, Berlin, Germany), according to the protocol described by the manufacturer: a total of 50 µL of aqueous supernatant was put in a micro-test tube, and the freezing point of the sample was measured. 

All reagents used in the biochemical analyses were purchased from Merck KGaA, (Darmstadt, Germany).

### 4.5. RT-PCR Analysis of T. aestivum Genes Coding Enzymes from L-Proline Biosynthesis Pathway

GeneJET Plant RNA Purification Kit (Thermo Scientific, Waltham, MA, USA) was used for total RNA extraction and purification. The concentration of the RNA obtained from each sample was determined on a micro-UV–VIS spectrophotometer, Nano Drop 2000 (Thermo Scientific, Basel, Switzerland). Copy DNA was synthesized from 1 μg total RNA using Scriptase RT—cDNA Synthesis Kit (GENAXXON Bioscience, Ulm, Germany) following the manufacturer’s instructions. The expression of gene transcripts of *T. aestivum* delta-1-pyrroline-5-carboxylate synthase (*P5CS*, LOC606368) and pyrroline-5-carboxylate reductase (*P5CR*, LOC606347) was evaluated by quantitative real-time PCR (qRT-PCR) using 2X GreenMasterMix No ROXTM (GENAXXON Bioscience, Ulm, Germany) and primers added at a final concentration of 0.1 µM in a ‘PikoReal’ Real-Time PCR System (Thermo Scientific, Basel, Switzerland). The qPCR analysis was conducted as follows: initial denaturation at 95 °C for 15 min; 45 cycles of 95 °C for 15 s (denaturation), 60 °C for 30 s (annealing) and 72 °C for 30 s (extension); and a final extension at 72 °C for 2 min. A melting curve analysis of the PCR products was performed at 60 °C–95 °C in 0.2 °C increments for 60 s to verify that the fluorescence detected during the run came from a single amplicon. The expression was normalized using the reference genes coding for alpha tubulin (U76558.1), 18S ribosomal RNA (LOC123171822) and elongation factor-1 alpha (LOC123123039). The calculation of the expression levels was performed according to the ΔΔCq method described in [58].

The primer pairs (Table 1) used in the analyses were designed with the Primer-BLAST tool available at the National Center of Biotechnology Information database (NCBI) (https://www.ncbi.nlm.nih.gov/tools/primer-blast/index.cgi accessed on 20 March 2021).

### 4.6. Statistical Analysis

The data presented were obtained from three separate biological experiments conducted in triplicate for biochemical and molecular assays and in ten replicas for biometrical parameters. The data represent average values with standard error (SE). One-way ANOVA with Duncan’s post hoc multiple range test were used to assess significant differences at the 0.05 level. All calculations were performed in MS Excel 2016 with the XLSTAT add-in.

## Figures and Tables

**Table 1:** Primer pairs used in the qRT-PCR analyses.

Gene Name	Locus	Forward Primer (5′-3′)	Reverse Primer (5′-3′)
*P5CS*	LOC606368	ctctacagcggtccaccaag	caggtacaccacccgttgaa
*P5CR*	LOC606347	taaatgccgttgttgctgcc	agcaaaactaacaatggctaccag
*α-TUB*	U76558.1	ttctcccgcatcgaccacaagttt	tcatcgccctcatcaccgtcc
*18S RNA*	LOC123171822	tacctggttgatcctgccagt	caatgatccttccgcaggttcac
*EF-1 α*	LOC123123039	cagatcggcaacggctac	gagaaggtctccaccaccat

## Data Availability

Research data are available upon request from the corresponding author.
